# Whole Breast and Lymph Node Irradiation using Halcyon™ 2.0 Utilizing Automatic Multi-isocenter Treatment Delivery and Daily Kilovoltage Cone-beam Computed Tomography

**DOI:** 10.7759/cureus.4744

**Published:** 2019-05-24

**Authors:** Michele M Kim, Chris Kennedy, Ryan Scheuermann, Gary Freedman, Taoran Li

**Affiliations:** 1 Radiation Oncology, Perelman School of Medicine, University of Pennsylvania, Philadelphia, USA

**Keywords:** radiation therapy, whole breast irradiation, halcyon, flattening filter free, multi-isocenter

## Abstract

The Halcyon^TM^ is a newly introduced platform to provide a simplified high-throughput workflow. A substantial fraction of patients treated with radiation are receiving breast irradiation. The Halcyon has a smaller maximum field size (28 x 28 cm^2^) compared to traditional C-arm linacs, limiting treatment of larger breast fields. With the use of autofeathering and linked multiple isocenters, non-divergent beams can be used to treat whole breast with large mid-tangent separation. In this case report, a multiple isocenter whole breast treatment with nodal involvement is described with the Halcyon 2.0 platform. The patient was simulated with the same immobilization techniques as used for C-arm linac treatments. The treatment planning time was around 20-30 minutes, which is similar to traditional planning for C-arm linacs, and dosimetric analysis resulted in satisfactory dose-volume histogram (DVH) parameters that met all planning objectives. Treatment times were shorter with an average of 9:32 minutes from the beginning of imaging to the end of treatment and a total in-room time of around 15 minutes per fraction. The use of multiple isocenters for the extended treatment field created a half-beam block type field arrangement, which has been previously reported to produce superior dosimetry results at the supraclavicular-tangents junction area

## Introduction

Whole breast radiation therapy after lumpectomy is used to control breast cancer, the most prevalent non-skin cancer among women in the United States [1]. Early-stage breast cancer can be controlled with radiation as an alternative to mastectomy [2]. Post-operative radiation therapy reduces the risk of local recurrence and increases long-term survival compared to mastectomy or lumpectomy alone [3]. The most commonly used treatment planning technique for adjuvant radiation of the breast in the United States is the 3D field-in-field technique using tangential fields on a C-arm linac [3-5]. The tangent field technique is efficient, simple, and effective for creating a uniform dose distribution within the breast treatment volume.

Halcyon^TM^ (Varian Medical Systems, Palo Alto, CA, USA) is a newly introduced treatment delivery platform that is designed to have efficient, high-throughput, simplified workflow. The Halcyon^TM^ is a closed system with a 100 cm diameter bore opening and a single 6 MV flattening filter-free (FFF) beam with a maximum field size of 28 x 28 cm^2^. Daily image guidance is required on the Halcyon^TM^ as there is no light field nor an optical distance indicator (ODI).

Traditional breast treatment plans on a C-arm linac is usually with 6 MV or 6 MV mixed with higher energies without daily imaging as the patient setup is performed with field light agreement with skin marks and source-to-surface distance (SSD) confirmation. Daily orthogonal kilovoltage imaging setup is also common as it provides more advantages than the cost of exposure to the patient. Furthermore, extended treatment fields can be treated with a single beam isocenter due to the larger maximum field size as compared to Halcyon^TM^.

We have previously reported a case on whole breast radiation therapy on Halcyon^TM^ without nodal involvement [6]. For Halcyon^TM^ platform, treating the whole breast and the involved nodal area was challenging due to the limited longitudinal field size of 28 cm, or 14 cm from isocenter. To allow for precise beam matching between the supraclavicular and tangent fields and minimize hot or cold spots due to beam divergence, isocenter for the tangential fields treating the breast is traditionally placed at the superior edge of the breast treatment fields, which leaves only 14-cm field size to cover the breast tissue if using Halcyon^TM^. For patients with relatively large breast extending beyond 14 cm in the superior-inferior direction, a single isocenter at the supraclavicular-tangents match line is not sufficient to cover the entire breast tissue.

This case report describes an experience with administering breast irradiation with Halcyon^TM^ for a large volume with three isocenters to treat the whole breast and involved nodes. The whole breast irradiation was performed with two isocenters to cover the entire volume with four total tangent fields.

## Case presentation

Patient workup and prescription dose

The patient was a 61-year-old woman with left breast cancer in the lower inner quadrant (7:00). She was treated post-chemotherapy. Pathology showed an AJCC (The American Joint Committee on Cancer) stage IA, ypT1a pN1mi cM0. She presented with left breast skin dimpling in February 2018 with an underlying palpable abnormality. A mammogram on February 14, 2018 showed skin retraction in the left lower inner quadrant with asymmetry deep within the lower left breast. A core biopsy of the left breast on February 15, 2018 showed invasive ductal carcinoma, poorly differentiated, positive lymphovascular invasion (LVI), associated with intraductal carcinoma (comedo type, European Organisation for Research and Treatment of Cancer (EORTC) high grade). A core biopsy of the suspicious left axillary lymph node showed metastatic ductal carcinoma that was estrogen receptor positive (ER+), progesterone receptor positive (PR+), and human epidermal growth factor receptor 2 positive (HER-2+). She received anastrozole as anti-estrogen hormone therapy.

She completed neoadjuvant TCHP (Taxotere + carboplatin + Herceptin + Perjeta) chemotherapy on June 22, 2018.

A partial mastectomy on July 24, 2018 revealed infiltrating poorly differentiated duct carcinoma with micropapillary features with the residual tumor having a largest dimension of 0.4 cm and extensive lymphatic invasion. Margins were free of tumor. Left sentinel lymph node (SLN) biopsy with two out of six nodes with micrometastatic carcinoma (ER+, PR+, HER-2+). Left axillary node dissection on August 9, 2018 demonstrated 0 out of seven lymph nodes involved by tumor.

Treatment prescription was 200 cGy x 25 fractions = 5000 cGy to the whole breast and supraclavicular/axillary/internal mammary nodes using tangent fields. Boost to the surgical bed 200 cGy x 5 fractions = 1000 cGy.

CT simulation and patient positioning

The patient was oriented in the head-first supine position on the Qfix angle board (Qfix, Avondale, PA, USA) with arm shuttle. Both arms were extended with hands grasping the arm shuttle’s poles behind the patient. The breast board angle was set to 10 degrees to level the sternum. The patient’s arms and head were immobilized by a Vac-Lok bag. The patient was simulated under deep inspiration breath hold (DIBH) using the SDX device (Dyn’r Medical Systems, Aix-en-Provence, France).

Prior to the simulation, the physician placed wires to delineate the breast volume, surgical scar, and the longitudinal extent of the treatment volume. Fiducial markers were placed prior to the scan to define the setup isocenter approximately midway between the superior and inferior wires at the patient midline. Markers were placed anteriorly at midline, and both sides laterally at a location of relatively stable anatomy. Two additional markers were placed along the midline to assist with straightening at setup.

CT simulation scan extended from chin through the whole lung. The reconstructed slice thickness was 3 mm and the reconstruction field-of-view was 65 cm. The patient was marked at the locations of the fiducial markers mentioned above. Measurements of the contralateral elbow position relative to the CT table and patient midline were made by the simulation therapists to assess potential collision with the Halcyon bore.

Patient positioning, immobilization, and image reconstruction settings were all consistent with institutional standards for this type of treatment with the exception of the measurements to assess potential Halcyon bore collision.

Imaging modalities used for contouring

Contouring was performed on the CT simulation image. No additional image registrations were performed. This is consistent with institutional standards for this type of treatment. The planning target volume (PTV) structure was created in accordance with the contouring guidelines recommended by the Radiation Therapy Oncology Group (RTOG). A whole breast clinical target volume was first created by the physician, followed by a uniform 7-mm expansion to create the planning target volume.

Treatment plan dosimetric summary

**Figure 1:**
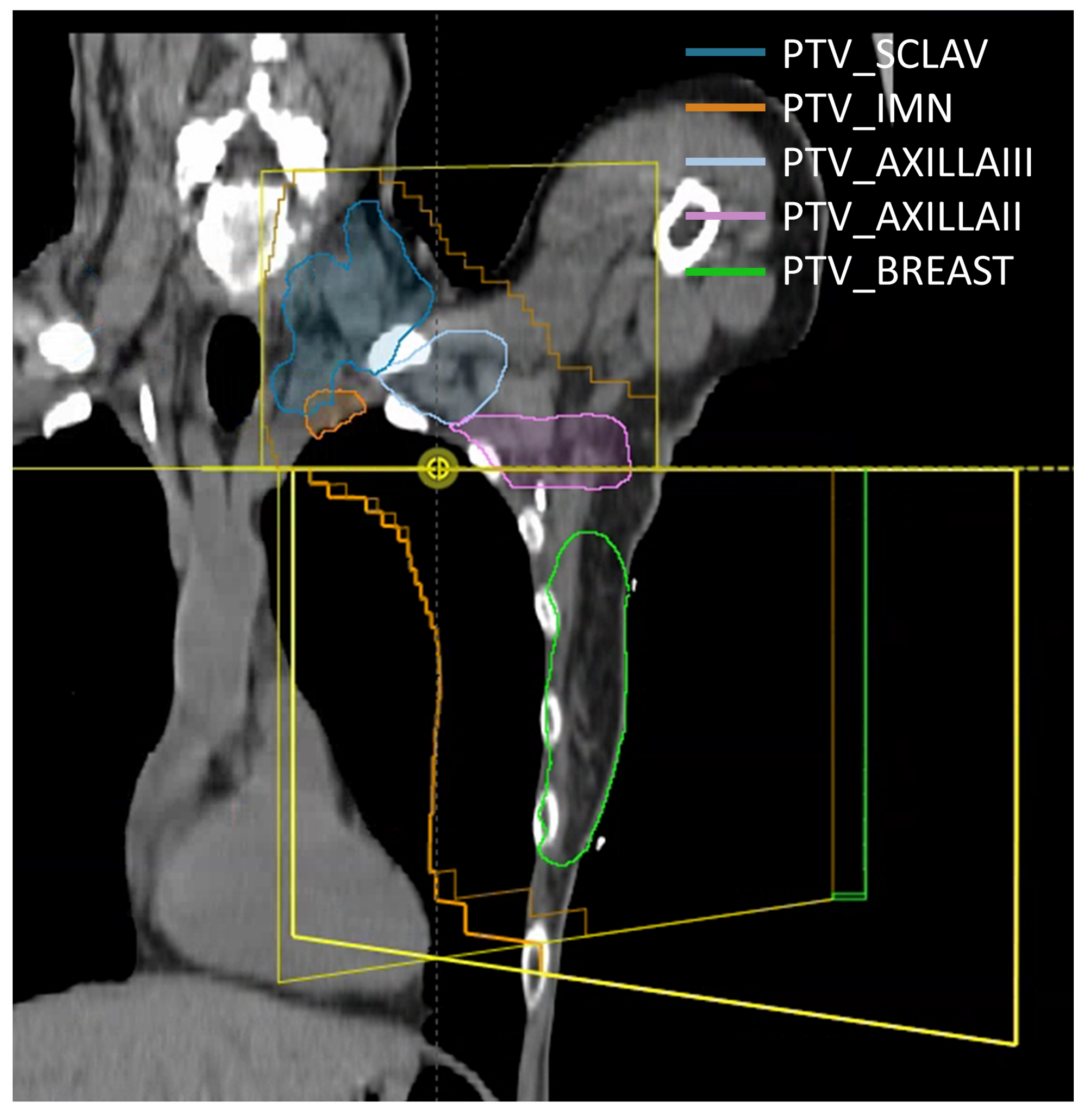
C-arm plan showing the fields defined by the physician PTV_SCLAV, supraclavicular planning target volume; PTV_IMN, internal mammary lymph node planning target volume; PTV_AXILLAIII, level III axillary lymph node planning target volume; PTV_AXILLAII, level II axillary lymph node planning target volume; PTV_Breast, breast planning target volume

Initial field placement was performed by the physician in Eclipse software using a C-arm machine model to set the gantry, collimator, and jaw positions to define the treatment extent. The fields were defined using a single isocenter with a half-beam block type technique for nodal breast treatment. The isocenter for the C-arm plan was chosen at the desired match line location for the tangent field/supraclavicular field border. The total length of the treatment field from the post-superior aspect of the supraclavicular fields to the most inferior aspect of the tangent fields was approximately 28 cm. Figure 1 shows the C-arm plan along with the target volumes (supraclavicular planning target volume - PTV_SCLAV; internal mammary lymph node planning target volume - PTV_IMN; level III axillary lymph node planning target volume - PTV_AXILLAIII; level II axillary lymph node planning target volume - PTV_AXILLAII; breast planning target volume - PTV_BREAST). This is in accordance with institutional standards for this process.

The planner used the C-arm linac fields to develop a multiple isocenter Halcyon plan using the following procedure:

1. Generate irradiated volume based on the physician’s beam and aperture settings. Calculate dose using the C-arm tangent fields defined by the physician and create a 50% isodose structure to define the tangent field irradiated volume.

2. Create a new Halcyon plan at the C-arm plan isocenter with supraclavicular/posterior axillary boost (PAB) fields with the same field apertures defined by the physician using the C-arm model. The collimator for the supraclavicular field is rotated to 90 degrees to allow for fine fluence adjustment at the supraclavicular-tangents match line.

3. Create an irregular surface compensator for the supraclavicular field using a 40% penetration depth.

4. Calculate the PAB field using a flattened field sequence, and adjust the weighting and fluence of the supraclavicular/PAB plan to achieve appropriate coverage of nodal volumes.

5. Create a new multiple-isocenter Halcyon plan with opposed tangent fields using the same gantry angles defined by the physician at the two isocenters: 1) one isocenter is placed at the supraclavicular/tangential match line defined by the physician in the C-arm plan, and 2) a second isocenter 8 cm inferior to the C-arm plan isocenter.

6. Optimize the new multi-isocenter Halcyon plan with auto-feathering enabled to achieve uniform dose to the irradiated volume isodose structure from Step 1. This step does not provide any modulation for the purpose of organ-at-risk (OAR) sparing, but is only used to creates an auto-feathered fluence between the two tangent field isocenters to deliver uniform dose within the irradiated volume defined by the physician.

7. Create a new combined plan with both the Halcyon nodal fields (supraclavicular/PAB) and tangent fields.

8. Edit the fluence of the nodal and/or tangent fields as necessary in the combined plan.

**Figure 2:**
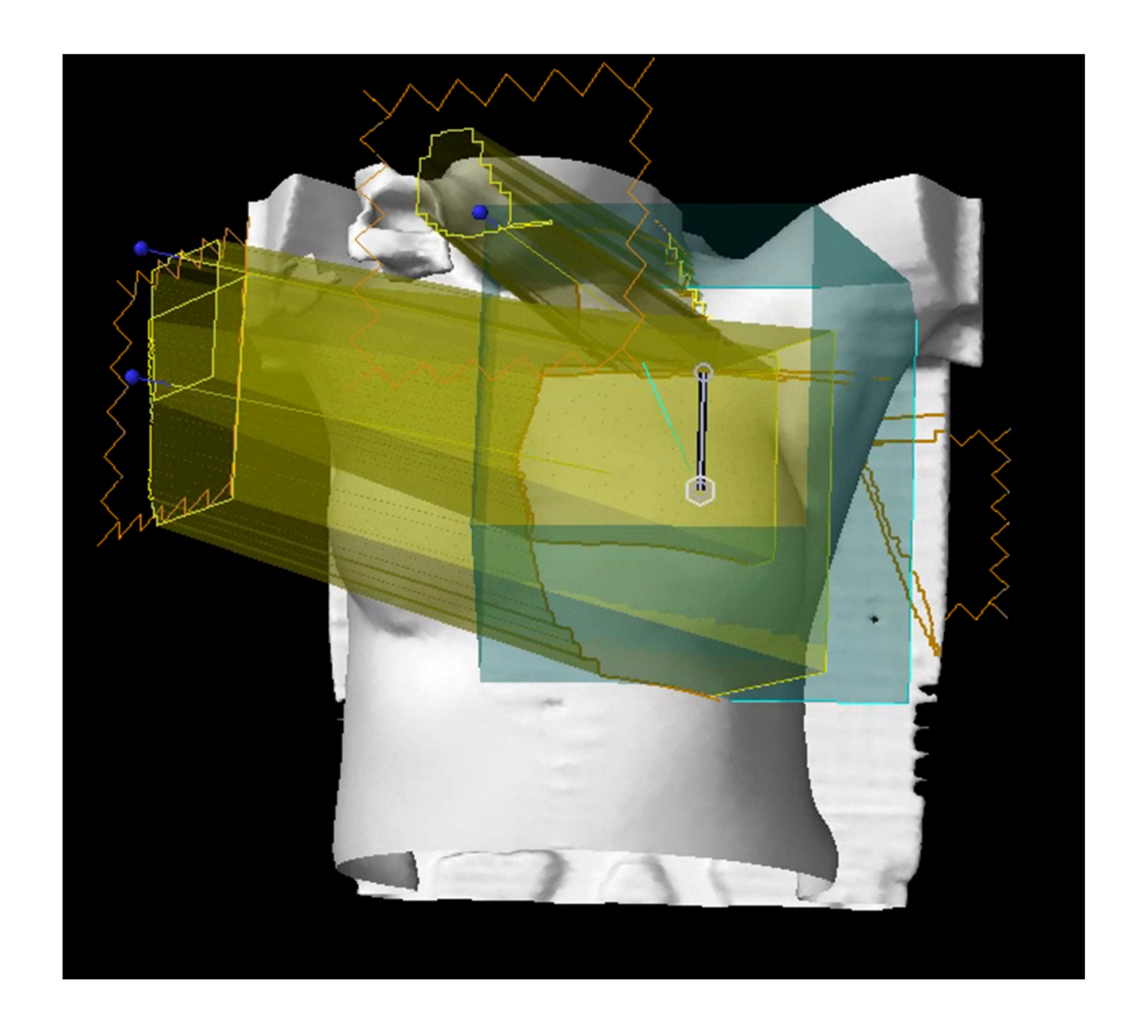
Projection of field arrangement (yellow) onto BODY structure (white)

The projections onto the BODY structure of the resulting field arrangement are shown in Figure 2. Figure 2 also shows the linked isocenters with an 8 cm separation (grey line). The maximum separation between the isocenters allowed by the treatment machine to utilize automatic delivery without requiring re-imaging of the second isocenter is 8 cm. An isocenter difference larger than 8 cm is allowed by the autofeathering functionality but will require two different CBCTs, one per isocenter. The isocenter difference was selected to be at 8 cm or under to reduce the imaging dose.

Fluence editing was performed on tangent fields to achieve the following dose objectives listed in Table 1. All planning objectives were met for this case. PTVeval_BREAST is defined by cropping the BREAST_PTV structure by 5 mm from the skin surface.

**Table 1:** Treatment planning objectives DVH, dose–volume histogram; PTVeval_Breast, breast planning target volume cropped 5 mm from the skin surface; PTV_SCLAV, supraclavicular planning target volume; PTV_AXILLAIII: level III axillary lymph node planning target volume; PTV_AXILLAII: level II axillary lymph node planning target volume; PTV_IMN: internal mammary lymph node planning target volume

Target and Critical Normal Tissue Constraints					
Structure Name	Dose Volume Histogram (DVH) Objective	Evaluator	Variation Acceptable	Priority	Achieved Plan Value
PTVeval_BREAST	D95%[Gy]	≥47.5	≥45	1	49.1
PTVeval_BREAST	V90%[%]	≥99	≥98	2	100
PTVeval_BREAST	V105%[%]	≤10	≤15	2	3.2
PTVeval_BREAST	Max[%]	≤107	≤110	1	107.2
Contralateral Breast	D5%[Gy]	≤2	≤3	3	5.2
Ipsilateral Lung	V20Gy[%]	≤30	≤35	2	29.7
Ipsilateral Lung	V5Gy[%]	≤65	≤70	2	47.7
Contralateral Lung	V5Gy[%]	≤10	≤15	3	0.21
Heart	V20Gy[%]	≤2	≤5	2	1.44
Heart	Mean[Gy]	≤3	≤4	2	1.94
PTV_SCLAV	D95%[Gy]	≥47.5	≥45	1	50.3
PTV_AXILLAIII	D95%[Gy]	≥47.5	≥45	1	47.5
PTV_AXILLAII	D95%[Gy]	≥47.5	≥45	1	46.3
PTV_IMN	D95%[Gy]	≥47.5	≥45	1	46.7

**Figure 3:**
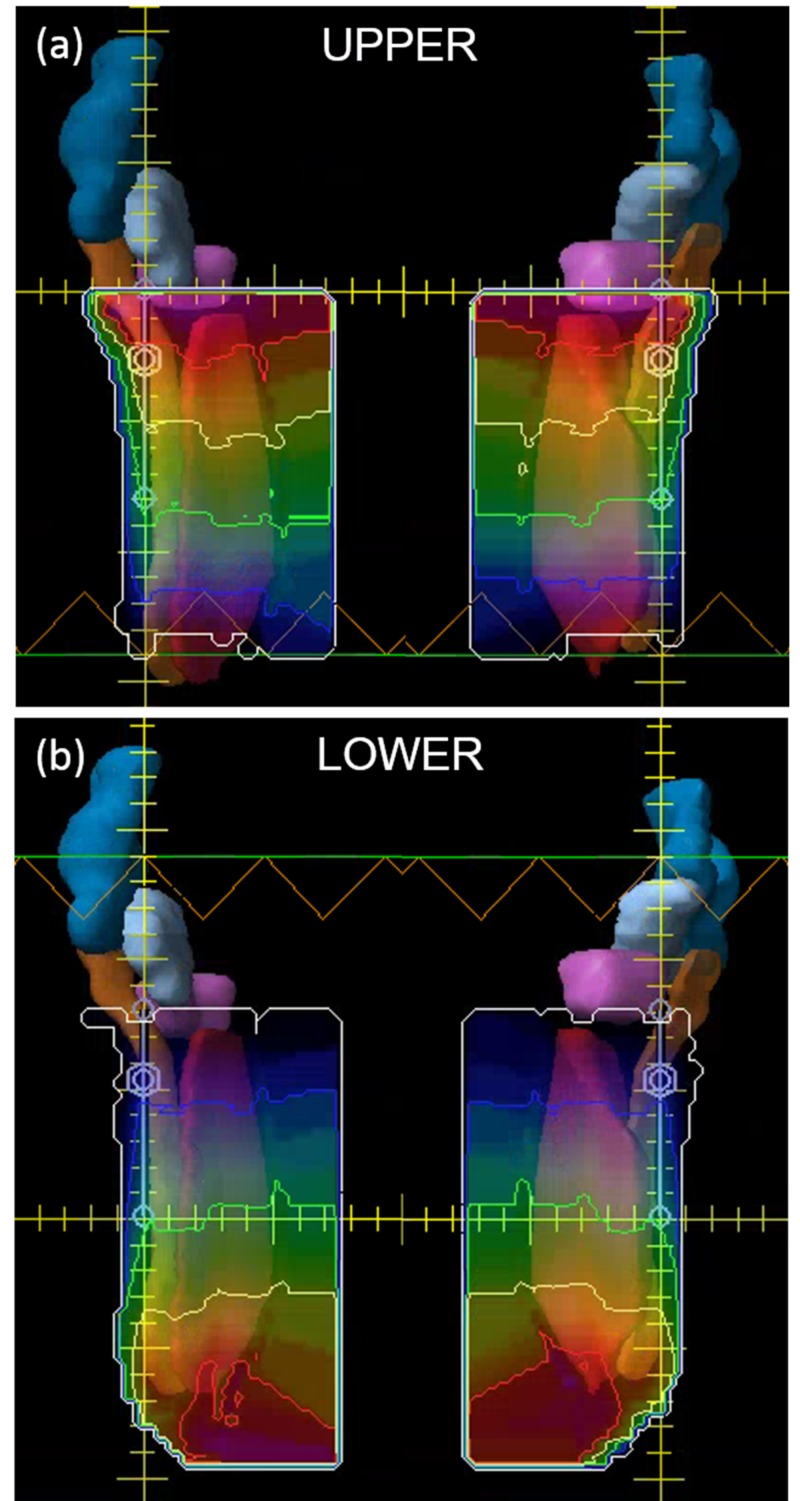
Fluence for the tangent fields showing the auto-feathered gradients for the medial and lateral tangent fields between the (a) superior and (b) inferior fields around the match line isocenter All PTV volumes are shown. Notice the gradient characteristics of the fluence maps used to achieve automatic feathering between the upper and lower isocenter fields. PTV, planning target volume

The resulting fluence for the tangent fields are shown in Figure 3. Skin flash was added to the fluence using the skin flash tool in Eclipse.

**Figure 4:**
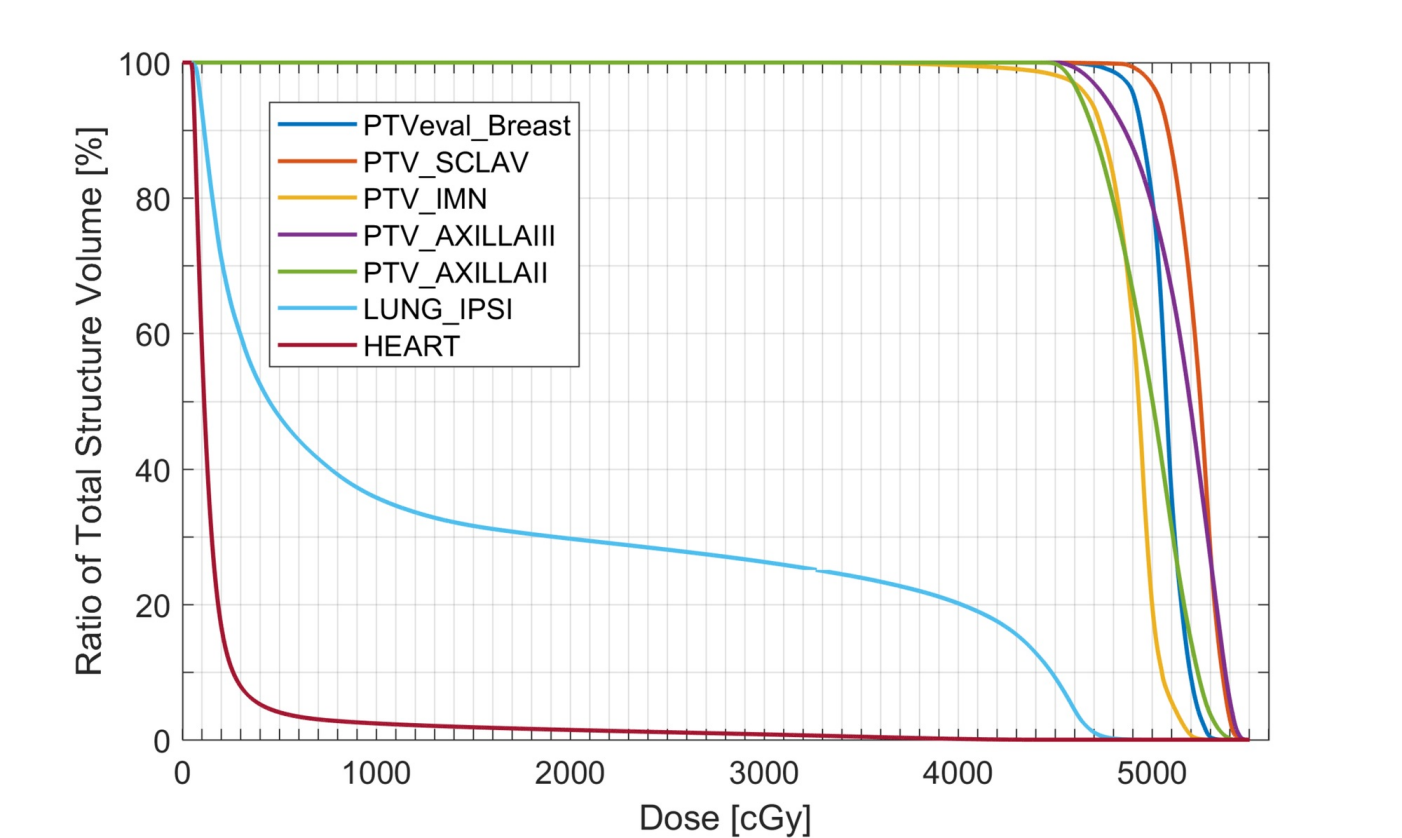
The DVH for the final plan DVH, dose–volume histogram; PTVeval_Breast:  Breast planning target volume cropped 5 mm from the skin surface; PTV_SCLAV, supraclavicular planning target volume; PTV_IMN, internal mammary lymph node planning target volume; PTV_AXILLAIII, level III axillary lymph node planning target volume; PTV_AXILLAII: level II axillary lymph node planning target volume; LUNG_IPSI: ipsilateral lung

The dose-volume histogram (DVH) for the plan is shown in Figure 4. The maximum dose for the plan was 110.2% of the prescription dose (5000 cGy), with the global maximum point occurring in the match line region. All planning objectives were satisfied.

Daily imaging selection

Kilovoltage cone-beam computed tomography (kV CBCT) images were used for daily localization of the initial fields. The imaging instructions for this case were to match the chest wall and to ensure that all of the breast tissue was included in the breast planning target volume (BREAST_PTV) structure and that the internal mammary lymph node planning target volume (IMN_PTV) structure is within the beam path.

kVCBCT image guidance

For daily positioning, an external sagittal laser was found to be beneficial for patient straightening due to the limited extent of the internal Halcyon sagittal laser. Reimaging was necessary for two out of the 25 treatments. 

kV CBCT image quality was excellent to visualize key anatomical structure for daily image-guided radiation therapy (IGRT), using a low-exposure breast technique (125 kV, 30 fps, 49 mAs, 16.6 s acquisition time) [7]. Fast acquisition enabled for complete CBCT acquisition under one breath-hold, which increased patient comfort and reduced the potential for imaging artifact associated with multiple breath-hold. Large field of view (24.5 cm longitudinal, 49.1 cm axial) provided sufficient visualization of key anatomical structures. Image dose for daily CBCT using breast protocol is relatively low compared to other CBCT protocols (e.g. thorax protocol with 301 mAs).

**Figure 5:**
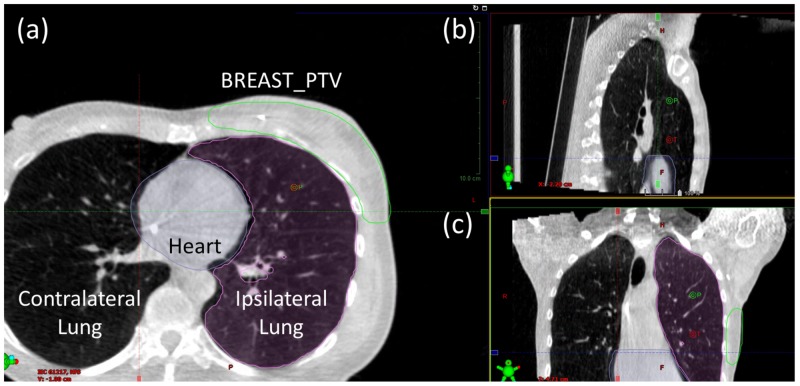
Breast protocol CBCT of tangent fields shown in the (a) axial, (b) sagittal, and (c) frontal views Overlays of the heart (purple), ipsilateral lung (pink), and BREAST_PTV (green) structures from the planning CT allow assessment of alignment quality. CBCT, cone-beam computed tomography

Alignment was performed for the chestwall, and it was verified that the breast, heart, and ipsilateral lung structures were in good agreement with the planning CT (Figure 5). The imaging isocenter was chosen to be able to visualization all anatomy features of interest, including chest wall, nodal areas, and the ipsilateral arm position. 

Treatment delivery summary

**Figure 6:**
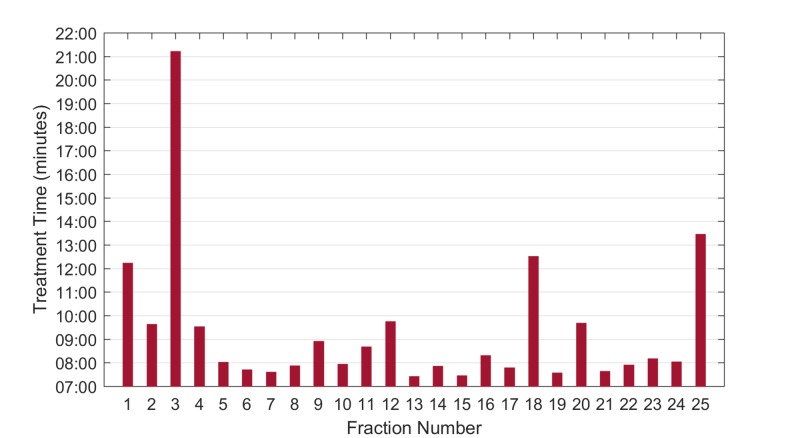
Treatment delivery times by fraction Extended time for fraction 3 was due to waiting for an available physician to approve CBCT images. CBCT, cone-beam computed tomography

The patient’s appointment length was around 15 minutes for each fraction except for fractions 1 and 3 for which the appointment length was 30 minutes. This additional time for these fractions is due to the necessity of physician approval of imaging prior to treatment. The daily combined imaging and treatment delivery time and total treatment room time are shown in Figure 6. kV CBCT images were used for daily localization of the initial tangent fields with instructions to match to the chestwall and to ensure that all of the breast tissue was included in the BREAST_PTV structure. The average time between the start of imaging and the completion of treatment was 9.32 minutes. This is longer than the average treatment time on Halcyon due to the use of DIBH with the SDX device.

The treatment time for fraction 1 was significantly extended due to an issue discovered at treatment. It was not possible to perform IGRT matching on the original CBCT. After investigation, it was discovered that the problem was caused by the treatment isocenters being located slightly more than 8 cm apart. It is recommended that when multiple isocenters are used for Halcyon, the isocenter shift of 8 cm is typed directly into the beam properties, as opposed to manually positioning, to prevent any rounding discrepancies to cause an undeliverable plan. The treatment time shown in Figure 6 does not reflect this delay.

## Discussion

**Figure 7:**
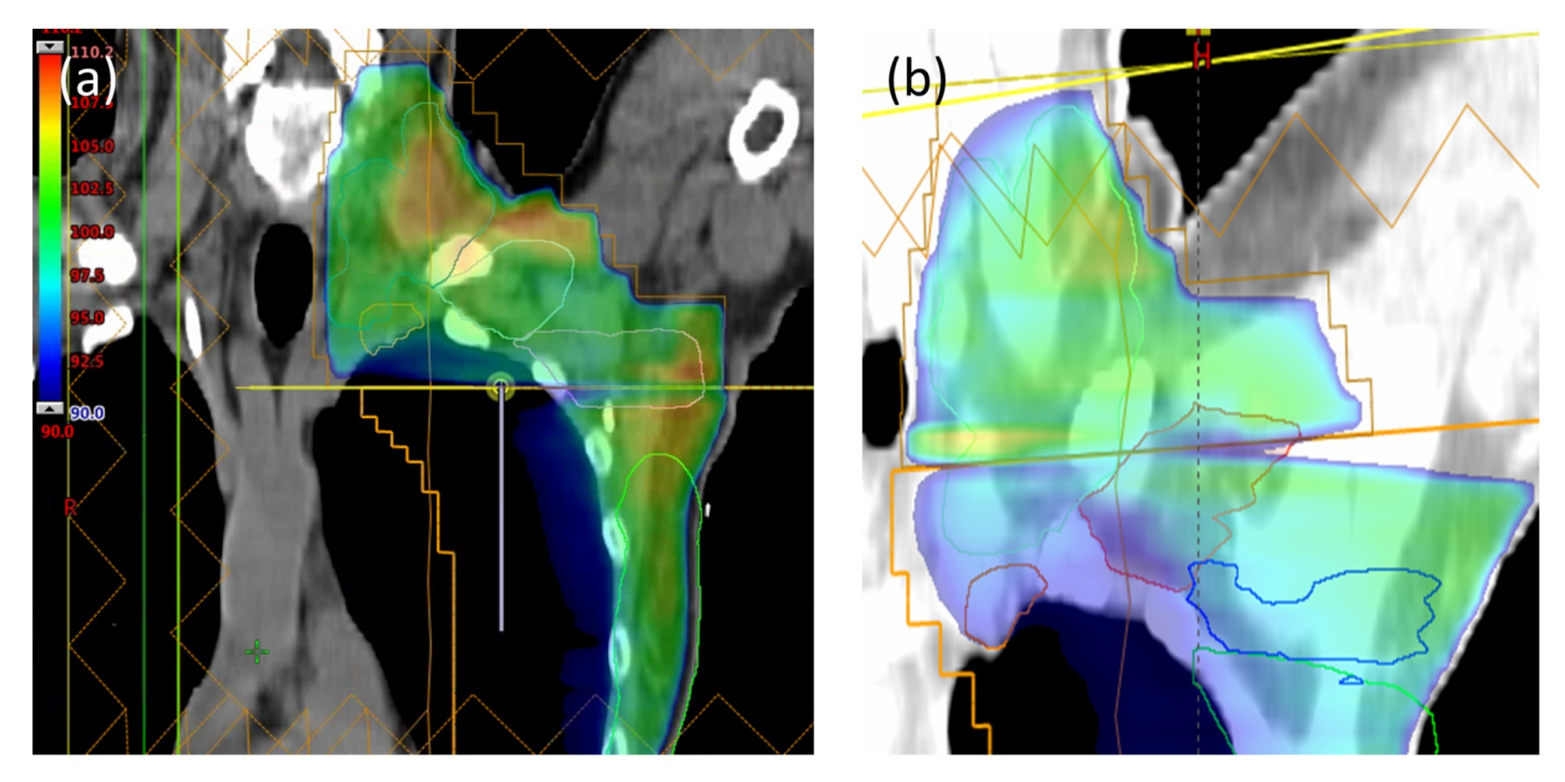
Comparison of dose distributions near the supraclavicular/tangent field match line for the (a) Halcyon 2.0 multiple isocenter plan and the (b) Halcyon 1.0 single isocenter plan for a prior case Note the hot and cold triangles that are present for the single isocenter plan due to beam divergence. These are not present in the Halcyon 2.0 plan due to the use of a half-beam block technique allowed by having multiple isocenters.

The use of multiple isocenters allows for a half-beam block type beam arrangement to be used at the match line isocenter for Halcyon 2.0 treatment plans. The use of this technique allows for an improved dose distribution compared to Halcyon 1.0 plans due to the lack of beam divergence at the match line. Figure 7 shows a comparison of the present plan’s dose distribution near the match line compared to a prior single-isocenter Halcyon 1.0 plan. As shown in the figure, the hot-cold triangles in the Halcyon 1.0 plan are not present in the Halcyon 2.0 plan owing to the use of the half-beam block technique that is possible due to the use of multiple isocenters. A comparison of the use of half-beam versus full-beam in breast radiotherapy by Kagkiouzis et al. concluded that the full-beam creates a non-satisfactory dosimetry result at the junction region, which can be mitigated by the half-beam technique [8].

**Figure 8:**
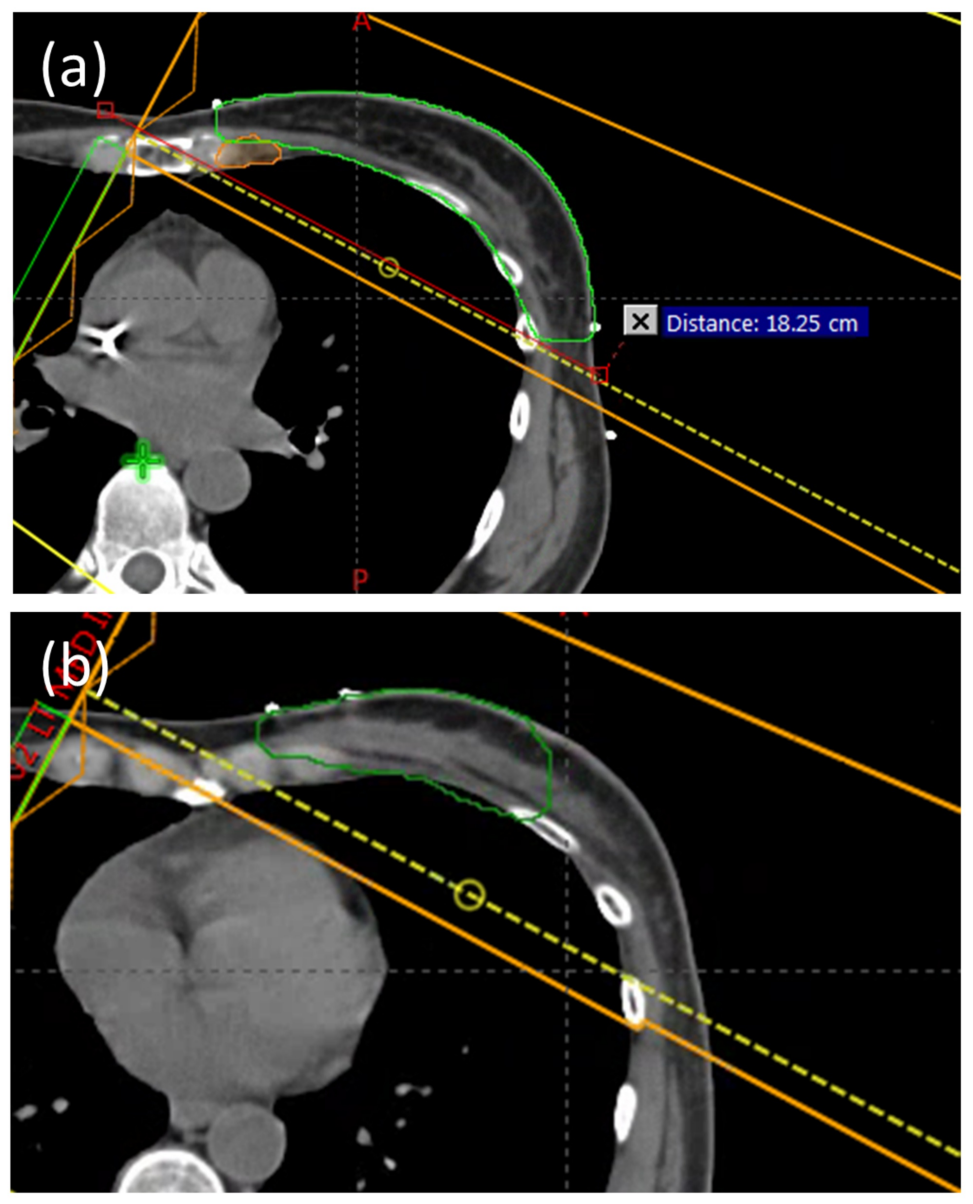
(a) Patient separation at mid-tangent, (b) Boost volume shown in green

The patient separation at mid-tangent measured to be 18.3 cm on the central field border (Figure 8a). The kV imaging isocenter for the INITIAL plan was chosen to be at the treatment isocenter near mid-breast at 8 cm inferior to the match line isocenter. Due to the size and location of the boost volume (Figure 8b), it was decided to treat the boost plan on a C-arm linac with electrons. The medial electrons and the shallow depth allowed easy access by an electron field with minimal heart dose while also sparing the lateral half of the breast. However, a tangent field arrangement on Halcyon would also likely have been an acceptable option. The planning time for this case was comparable to that of a C-arm linac single isocenter breast plan.

No clinical differences in skin reaction were noted relative to the institutional standard treatment with field-in-field technique on a C-arm linac with 6 MV/10 MV energies.

## Conclusions

Halcyon 2.0 with kV CBCT and two-isocenter automatic feathering and delivery enables accurate and faster treatments than with a conventional C-arm linac. The use of multiple isocenters and auto-feathered gradient field matching minimized beam divergence at the supraclavicular-tangent field match line and allowed for treating extended breast target with less hot and cold triangles compared to a single isocenter plan. Automatic shift and delivery between kV CBCT isocenter and two treatment isocenters minimized multi-isocenter’s impact on total treatment time while allowing for customized imaging range (scan length) setting. Overall, the simulation and planning process was similar to breast treatment planning on C-arm linacs. Delivery efficiency was increased due to the use of a 6 MV FFF beam energy and automatic shift-and-treat feature, and the total treatment time was drastically reduced.
